# Antimicrobial effect of biological surgical mesh added with vancomycin or silver nanoparticles for multidrug-resistant bacteria: experimental study in rats

**DOI:** 10.1590/0100-6991e-20253835-en

**Published:** 2025-12-23

**Authors:** MARCELO DE PAULA LOUREIRO, PIETRO MARAN NOVAIS, ISHMAEL THOMAZ PADILHA, MARINA SCHMID GUÉRIOS, FÁBIO LUIGI CRISIGIOVANNI, VICTORIA BIZZI VIEIRA, ANA LUIZA MASSELAI, RODRIGO MÜLLER CARAVALHO, EDUARDO SALAMACHA, LUANE ZONTTA, MARINA DA COSTA GOMES, ENRICO BERTOLUCCI BOSCARDIM, ISADORA UTRI ANDREGUETTO, FELIPE FRANCISCO TUON

**Affiliations:** 1 - Universidade Positivo, Pós-Graduação em Biotecnologia - Curitiba - PR - Brasil; 2 - Universidade Positivo, Graduação em Medicina - Curitiba - PR - Brasil; 3 - Pontifícia Universidade Católica do Paraná, Laboratório de Doenças Infecciosas Emergentes - Curitiba - PR - Brasil

**Keywords:** Rats, Wistar, Silver Compounds, Pericardium, Vancomycin, Antibiotic Prophylaxis, Ratos Wistar, Compostos de Prata, Pericárdio, Vancomicina, Antibioticoprofilaxia

## Abstract

**Introduction:**

Surgical site infection (SSI) and polypropylene mesh (PPM) infections are recurrent problems in abdominal hernia surgeries, highlighting the need for a new antimicrobial material for surgical repair. The aim of this study was to evaluate the in vivo antimicrobial effect of a new biological mesh made of decellularized bovine pericardium (BP), added with vancomycin (VAN) or silver nanoparticles (AgNPs), as prevention for SSI.

**Methods:**

Thirty-five Wistar rats were divided into four groups: BP C+ (n=9) with BP without additions; PP C+ (n=8) with PPM; BP AgNPs (n=9) with BP added with silver nanoparticles; and BP VAN (n=9) with BP added with vancomycin. The 1 cm² meshes were stitched into the muscle fascia under the subcutaneous tissue of the rats’ backs, followed by inoculation with methicillin-resistant Staphylococcus aureus. The animals were observed for 7 days, with subsequent euthanasia, and histological and bacteriological analysis.

**Results:**

The BP VAN group had better infection control compared to the PP C+ and BP AgNPs groups (1x10¹ vs. 1.4x10³CFU/g, p=0.0303; 1x10¹ vs. 1.5x10^4^CFU/g, p<0.0001, respectively). BP AgNPs showed less bacterial reduction compared to BP C+ (p=0.042). In the histological analysis, there was a mild inflammatory reaction in BP VAN, moderate in BP C+, and intense in PP C+ and BP AgNPs.

**Conclusion:**

BP added with vancomycin showed promising antimicrobial action, while the use of silver nanoparticles did not demonstrate efficacy in this study.

## RESUMO

**Introdução:**

A infecção do sítio cirúrgico (ISC) e das telas de polipropileno (TPP) é um problema recorrente em cirurgias de hérnias abdominais, evidenciando a necessidade de um novo material antimicrobiano para o reparo cirúrgico. O objetivo deste estudo foi avaliar o efeito antimicrobiano in vivo de uma nova tela biológica feita de pericárdio bovino descelularizado (PB), acrescida de vancomicina (VAN) ou nanopartículas de prata (AgNPs), como prevenção para a ISC.

**Métodos:**

Foram utilizados 35 ratos Wistar, divididos em quatro grupos: PB C+ (n=9) com PB sem acréscimos; PP C+ (n=8) com TPP; PB AgNPs (n=9) com PB acrescido de nanopartículas de prata; e PB VAN (n=9) com PB acrescido de vancomicina. As telas, com 1 cm², foram fixadas na fáscia muscular sob o subcutâneo do dorso dos ratos, seguidas de inoculação com Staphylococcus aureus resistente à meticilina. Os animais foram observados por 7 dias, com posterior eutanásia, análise histológica e bacteriológica.

**Resultados:**

O grupo PB VAN teve melhor controle da infecção em comparação aos grupos PP C+ e PB AgNPs (1x10¹ vs. 1,4x10³UFC/g, p=0,0303; 1x10¹ vs. 1,5x10^4^ UFC/g, p<0,0001, respectivamente). O PB AgNPs apresentou menor redução bacteriana em comparação ao PB C+ (p=0,042). Na análise histológica, houve leve reação inflamatória no PB VAN, moderada no PB C+, e intensa no PP C+ e PB AgNPs.

**Conclusão:**

O PB acrescido de vancomicina apresentou ação antimicrobiana promissora, enquanto o uso de nanopartículas de prata não demonstrou eficácia neste estudo.

## INTRODUCTION

Abdominal wall hernias are the most common surgical disease in General Surgery^1^. Data from the Brazilian Public Unified Health System (SUS) indicate that, between January 2023 and September 2024, open and laparoscopic inguinal hernioplasties totaled 330,568 procedures, which results in an average of more than 15,000 monthly procedures across the country^2^.

Most surgical techniques require the interposition of a synthetic mesh, especially polypropylene (PPM)^3^. For each patient, the characteristics of material manufacturing and mesh preparation should be chosen based on the possibility of hernia recurrence, local complications, and occurrence of infections. The main material classes used in the manufacture of surgical meshes are synthetic and biological. Within each, there are variations that affect tissue incorporation, tensile strength, and resistance to infection^4^.

The causes of surgical site infection (SSI) are diverse and vary according to the procedure. In general surgery, factors such as prolonged hospital stay and surgery duration directly affect the chances of patients developing infection, and the infection rate of hernia repair with the use of mesh can reach up to 12.4%^5^, the main etiological agents being *Staphylococcus aureus* and *Escherichia coli* ^*6*^.

Regarding SSI, microorganisms can form biofilm on synthetic meshes, leading to mesh explant due to inefficacy of the immune system and drugs, as well as increased risk of postoperative complications^4^. Therefore, synthetic meshes are contraindicated in contaminated environments, highlighting the necessity for alternative materials that can withstand infection and thereby minimize complications^7^. These complications can impair the integrity of the surgical mesh and patient’s health, since the infection can spread hematologically or cause intense local damage due to acute or chronic inflammation. In this setting, infection prophylaxis and proper preparation of materials to be utilized are essential.

Biological meshes are tissues treated through decellularization processes, serving as a foundation for neovascularization and cell proliferation processes^7^ ^,^ ^8^. However, such meshes are relatively expensive, and there are no protocols for the indication of this material9. This is because even though the basic composition is a collagen matrix, the fabrics produced vary in biomechanical aspects, biological compatibility, and ability to resist infection^8^. Therefore, it is important for surgeons to know and understand the profile and risks associated with each type of material, to increase procedures safety and efficiency. However, a factor that limits this choice is the lack of data that can be used to compare the materials used in the production of such meshes^10^. Bovine pericardial biological meshes, such as Tutomesh^®^ (decellularized bovine pericardial [BP] biological mesh), which is used for hernia repair or post-incisional hernia in a potentially contaminated surgical site, can reduce the risk of recurrence in the short term without increasing overall comorbidity^11^.

Due to the importance of SSI prophylaxis, vancomycin (VAN) is commonly used to target gram-positive bacteria, including resistant strains like methicillin-resistant *Staphylococcus aureus* (MRSA). Kraft et al.^12^ demonstrated that decellularized grafts containing Vancomycin showed antimicrobial activity for *Staphylococcus aureus* and *Bacillus subtilis*. In addition, the study evaluated the concentration of the antibiotic in the meshes at intervals of 0, 6, 12, and 18 months, showing good zones of growth inhibition of *Staphylococcus aureus* around the decellularized tissues plus vancomycin^12^.

Silver nanoparticles (AgNPs) represent an additional prophylactic approach, leveraging their distinct chemical and physical characteristics to address antimicrobial resistance in antibiotic therapy. AgNPs with the size of 10-100nm have shown strong antimicrobial effect against Gram-positive and negative bacteria^13^. This is possible by preventing the bacterial formation of biofilm, decreasing the absorption and increasing the efflux of the drug from the microbial cell^14^. In combination with antibacterial agents, such as organic compounds or antibiotics, AgNPs have demonstrated a synergistic effect against pathogenic bacteria such as *Escherichia coli and Staphylococcus aureus* ^15^. To promote greater adaptability of biological meshes (biomeshes), the use of AgNPs is a viable and promising alternative.

Another factor to consider is the cost of surgical meshes. In the short term, synthetic meshes have proven to be effective and cheaper. However, biosynthetic and biological fabrics, even at a higher cost, can be advantageous in the long run.

Despite the growing variety of surgical meshes and considerations of cost-effectiveness, there is currently no mesh suitable for all clinical scenarios. Additionally, comprehensive and comparative knowledge of the mechanisms of action of different meshes - particularly concerning recipient response and integration - remains limited^4^ ^,^ ^7^ ^,^ ^16^.

Considering that *Staphylococcus aureus* is the most found agent in mesh infections in hernia repair surgeries^16^, the great potential of using a biomesh plus antibiotics in the treatment of this microorganism becomes evident.

## METHODS

Since this is a research on animals, we submitted this work to the appreciation of the Ethics Committee on the Use of Animals (CEUA) of the Positivo University (UP), as determined by Law No. 11,794/08 and the resolutions of the National Council for the Control of Animal Experimentation (CONCEA), being approved on March 20, 2023, by opinion 3/2023, and following the ARRIVE checklist (Animal Research: Reporting of In Vivo Experiments)^17^.

The procedure for preparing the mesh from bovine pericardium (BP) involves a technique with lower cost, with the material originating from the post-mortem removal from animals for human consumption (from a local slaughterhouse) and its cleaning carried out manually. Aiming at the microbiological control of the sample, the decontamination of the mesh is done in an antibiotic solution containing Cefoxitin, Lincomycin, and Polymyxin B. Subsequently, its decellularization is performed in a detergent and chelating solution, with subsequent washing for 10 days with 70% ethanol (v/v) and 0.9% saline solution. Greater details of BP production and impregnation with VAN or AgNPs have been previously described^18^.

The determination of sample size considered a α error of up to 0.05 (5%), sampling power of 1-β error (20%) and using effect size based on previous experiments in this line of research, thus respecting the “3 Rs”.

The MRSA used was obtained from a wound of a patient admitted to the Cajuru University Hospital (Curitiba, Paraná, Brazil). The bacteria were cultured, collected, and resuspended in a glass tube with saline solution and homogenized in a vortex shaker, until reaching a standard solution of 0.5 McFarland (concentration of approximately 1.5 x 10 ^8^ CFU/mL [colony forming unit]). Then, the suspension was diluted at 1:50 in saline solution to obtain a new solution with a concentration of 3 x 10 ^6^ CFU/mL.

We used 35 female Wistar rats (*Rattus norvegicus albinus*, Rodentia mammalia, Wistar lineage) at 17 weeks of age and with an average weight of 257.85g±42.85, provided by the Vivarium of Positivo University, and randomly assigned to the following groups: 9 rats with BP placement without antibiotics (BP C+); 8 rats with PPM placement (PP C+); 9 rats with BP placement with Vancomycin (BP VAN); and 9 rats with BP placement with AgNPs (BP AgNPs). All the meshes used were square, measuring 1 x 1cm.

Subcutaneous morphine 0.1mg/kg was injected preoperatively. The surgical procedure was performed according to the following steps: (1) sedation (isoflurane in a glass bell) and anesthesia in the animal with intraperitoneal injection (ketamine 90mg/kg with xylazine 10mg/kg); (2) trichotomy of the dorsal region; (3) asepsis and antisepsis with 70% alcohol and PVPI; (4) apposition of sterile fields; (5) right and left longitudinal scapular-subscapular incision, 4 cm; (6) hydration of the mesh in the groups corresponding to the use of pericardium in 0.9% saline solution; (7) blunt subcutaneous dissection and implantation of the corresponding group mesh at the incision site over the muscle fascia; (8) application of 2 simple stitches with 5.0 nylon thread in 2 diagonally opposite vertices of the mesh, opposite to each other, fixing the material; (9) Contamination of the site with MRSA, inoculating 1 x 10 ^5^ CFU with 50 μL of the solution in all groups; (10) hemostasis revision; (11) Closure of the incisions using 5-0 nylon thread in simple sutures.

For postoperative and follow-up, the animals were placed under standard conditions, with adequate food for the rat diet and water, offered ad libitum. The lighting was produced by artificial light, turned on at 7 am and off at 7 pm. The animals were kept in standardized boxes with plastic bottoms and wood shavings, closed at the top with metal bars. Each of the boxes housed 2-3 rats, arranged according to the group. The environment had a stable temperature, with air exhaustion. The animals were kept for 10 days in these conditions, prior to the surgical act, to accommodate themselves to the environment. Analgesia was administered postoperatively to manage pain and minimize stress, following the Vivarium protocol, with assessments every 12 hours and rescue medication provided if pain was observed^19^. Postoperative follow-up lasted seven days. If sepsis occurred or pain did not respond to rescue medication, the rat was euthanized and excluded from the study.

After seven days, euthanasia was performed with an overdose of isoflurane by a veterinarian, under the guidance of the CONCEA euthanasia practice guidelines - resolution No. 37 of the Ministry of Sciences, Technology, Innovation, and Communication (Brasília/DF) of February 22, 2018. The wound was inspected to identify dehiscences and secretions, and subsequently, a median dorsal longitudinal incision was made with skin eversion to expose the meshes.

The materials were collected in 2 ways: 1) removal of the mesh only; 2) en bloc excision of the skin with the mesh, tissues with fibrosis, and/or adjacent abscess, in about 2 x 2cm. For each rat, it was randomly decided which side would be collected by forms 1 and 2.

For microbiological analysis, the material was removed and immediately placed in a 15ml tube containing 10mL of 0.9% saline solution. Then, two dilutions of 1:100 were made, both sterile. That is, after the process in the vortex shaker, 100μL of the pure solution was removed, which was placed in 10mL of 0.9% saline solution; this mixture was vortexed and again 100μL of the solution was removed and diluted in 10mL of 0.9% saline solution, being vortexed. This way we obtained pure solutions, one at 10 ^2^ and another at 10 ^4^ . Then, 100μL of each of the three obtained solutions were dispensed on TSA plates and seeded along the entire length with the aid of a Drigalski loop. The plates were incubated at 35°C ± 2°C for 20-24 hours. The number of colonies obtained on each TSA plate was manually identified. The result of the colony count was expressed in log^10^ of CFU/ml.

For histological analysis, the meshes were removed by form 2 and fixed in 10% formalin in sterile vials for 24 hours, with subsequent preparation of paraffin blocks. Four longitudinal cuts of 3mm in length were made containing the central region of the specimen, with the corresponding mesh and surrounding tissues. Tissue samples were fixed in 10% buffered neutral formaldehyde. Subsequently, the samples were embedded in paraffin and 4μm thick sections were obtained. To evaluate the morphological integrity of the meshes, the histological sections were stained with Hematoxylin and Eosin - H&E (Harris Hematoxylin: NewProv, Cód. PA203, Paraná, BR; Eosin: BIOTEC Analytical Reagents, Cod. 4371, Paraná, BR). The slides were scanned using an Axio Scan.Z1 slide scanner (Zeiss, Jena, Germany) and subsequent processing and analysis of the images were performed using Zen lite software (Zeiss, Jena, Germany).

The main outcome was the count of the number of colonies, presented by median and interquartile range (IQR) in colony-forming units per milliliter (CFU/mL), and compared with the nonparametric Kruskal-Walli’s test and with post hoc Dunn’s test for multiple comparisons. The histological analysis was performed in a semiquantitative manner, as described by Vizzoto et al.^20^, grading the presence of neutrophils, edema, congestion, monomorphonuclear cells, granulation tissue, and fibrosis according to intensity (marked, moderate, mild, or absent). Due to the observation time, we did not analyze the inflammatory process phase. 

Values of p<0.05 were considered significant after Bonferroni adjustment. The software used was the Prism 7.0 software (Graphpad, San Diego, CA, USA).

## RESULTS

Two rats in the BP VAN group were removed due to death two hours after the procedure.

**Figure 1:**
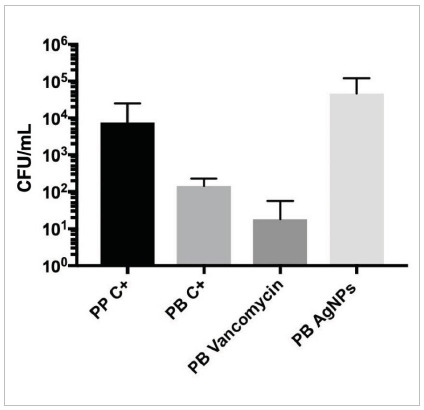
Quantitative analysis of colony growth on membranes in CFU/mL expressed on a log10 scale.

Regarding microbiological analysis, Figure 1 shows the analysis of colony growth. The medians, IQRs, and statistical analysis are shown in Table 1. In the BP VAN group, there was a greater bacterial reduction than in the PP C+ and BP AgNPs groups (p=0.0303 and p<0.001, respectively). Bacterial growth was similar between the BP C+ and PP C+ groups (p=1.0). There was less bacterial reduction in the BP AgNPs group compared with the BP C+ group (p=0.042). The BP meshes added to AgNPs showed a higher number of colony growth (1.5 x 10 ^4^ CFU/mL g) when compared with the other groups (vs. BP VAN, p<0.001; vs. BP C+, p=0.042; and vs. PP C+, p=0.4418), though with a reduction in initial inoculation of 1 x 10 ^5^ CFU/mL.

**Table 1:** Medians and confidence intervals.

Group	Meshes rated (n)	Median (CFU/mL)	IQR (Bottom Quartile - Top)	p-value^a^		
				vs. PP C+	vs. BP C+	vs. BP AgNPs
BP VAN	7	1 x 10 ^1^	0 - 1.8 x 10 ^1^	0.0303*	0.3481	<0,0001*
BP AgNPs	9	1.5 x 10 ^4^	1 x 10 ^3^ - 5 x 10 ^4^	0.4418	0.042*	-
BP C+	9	2 x 10 ^4^	1.7125 x 10 ^4^ - 2.3675 x 10 ^4^	1.0	-	-
PP C+	8	1.4 x 10 ^3^	1 x 10 ^2^ - 1.625 x 10 ^4^	-	-	-

^a^ Dunn’s test, post hoc test of the Kruskal-Wallis test. *Significant (p<0.05).

**Figure 2:**
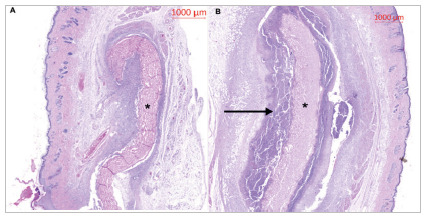
A) Photomicrograph of the central region stained with H&E containing pericardial mesh with vancomycin at lower magnification. * = Decellularized bovine pericardium. B) Photomicrograph of the central region stained with H&E containing pericardial mesh with silver nanoparticles encapsulated in an abscess at lower magnification. * = Decellularized bovine pericardium. Horizontal arrow = Abscess.

In the histological analysis, it was possible to see the BP VAN and BP AgNPs meshes in the subcutaneous tissue, and in the BP AgNPs one could perceive abscess formation encompassing the mesh, a macroscopically perceptible characteristic in all animals in this group (Figure 2).

**Figure 3:**
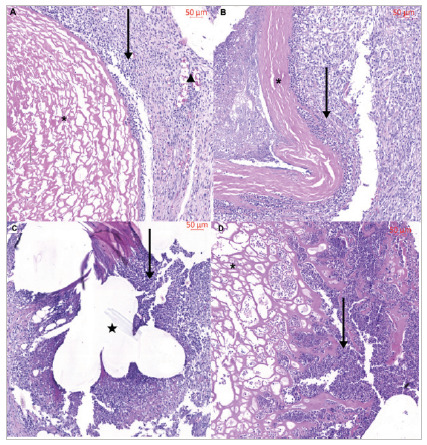
A) Photomicrograph of the central region stained with H&E containing pericardial mesh with vancomycin at higher magnification. * = Decellularized bovine pericardium. Vertical arrow = Inflammatory reaction. Triangle = Neovascularization. B) Photomicrograph of the central region stained with H&E containing pericardial mesh without antibiotic at higher magnification. * = Decellularized bovine pericardium. Vertical arrow = Inflammatory reaction. C) Photomicrograph of the central region stained with H&E containing polypropylene mesh at higher magnification. Star = Polypropylene mesh. Vertical arrow = Inflammatory reaction. D) Photomicrograph of the central region stained with H&E containing pericardial mesh with silver nanoparticles at higher magnification. * = Decellularized bovine pericardium. Vertical arrow = Inflammatory reaction.

In detail, mild neutrophilic inflammatory response and neovascularization were observed in BP VAN samples, while BP C+ exhibited a moderate reaction. PP C+ and BP AgNPs demonstrated intense responses, with the latter showing a notably greater loss of mesh collagen structural organization compared with BP C+ and BP VAN (Figure 3).

## DISCUSSION

The main agents responsible for post-surgical abdominal wall mesh infections are *S. aureus* (57.7%) (*Staphylococcus aureus*, which is sensitive to methicillin [MSSA] or MRSA) and *E. coli*. Therefore, it is important to know ways to avoid the infection itself or its more serious complications^6^. In a review, Falagas and Makris demonstrated a prevalence of up to 63% of MRSA as the etiology of surgical mesh infections used in hernias^21^, which points to the need for effective drugs against this type of condition. 

In this study, vancomycin was used as an antimicrobial, as it is known to fight MRSA infections^18^. The use of this intravenous antimicrobial does not present much benefit after the presence of infection, since the local response with the presence of the mesh is impaired due to the formation of a fibrotic capsule that hinders the action of vancomycin in humans. In addition, *Staphylococcus spp*. produces biofilm on the infected mesh, making it even more difficult for the immune system to fight the infection, rendering mesh removal part of treatment^22^. However, we observed a superior result of the use of vancomycin against MRSA bacterial development compared with the other groups tested; the action is also proven in MSSA infection^18^, showing that its local action can be effective in combating infection.

Regarding AgNPs, we found discrepancy of results between in vitro and in vivo studies as to antimicrobial action. A study by Ballo et al.^23^ showed that while in the in vitro environment antibiotics prevented more than 80% of infections, in the in vivo model infections were reduced by only 22%. Similarly, in the present study, BP meshes plus AgNPs showed less efficacy in containing infection. In addition, it is possible that AgNPs have cytotoxic action^18^, which, together with the loss of effectiveness, has contributed to the formation of abscesses. On the other hand, other pathogens also cause complications, and they can be avoided with the prophylactic use of silver, as shown in our previous study with MSSA^18^. Thus, dose adjustments and reductions of cytotoxic mechanisms may occur in further research. 

Regarding the use of biological meshes, the collagen base creates a foundation that gives structure and shape to guide the patient’s tissue regeneration, allowing better vascular infiltration and formation of new subcutaneous tissue, while undergoing a remodeling process^7^ ^,^ ^8^. These materials are already used in several types of surgery, since collagen has good biocompatibility, biodegradation, and absence of toxicity^7^ ^,^ ^8^ ^,^ ^24^ ^,^ ^25^. In this scenario, BP decellularization is indispensable. The method used to decellularize our meshes proved to be effective, as the absence of nuclei in BP was noticeable (Figure 3)^18^ ^,^ ^26^.

Thus, we observed antimicrobial action in the vancomycin mesh, as well as a good integration to the animal’s subcutaneous tissue, and the occurrence of neovascularization, with a lower local inflammatory process, as shown by the minimal infiltration of neutrophils.

In view of the various options for biological meshes, literature is usually conflicting about their indications in relation to PPM. This is due both to the quality of randomized controlled trials (RCTs) and to the parameters used^27^. Meta-analyses tend to group biological meshes with biosynthetic meshes in comparison with PPM, producing biased results^28^ ^,^ ^29^.

As for RCTs with biological meshes from BP, the mesh from Balance Medical, Ltd. (Beijing, China) used in inguinal hernias displayed no difference in recurrence or inflammation compared with PPM and showed a lower rate of pain up to three months postoperatively^30^. Another RCT showed that Tutomesh^®^ had lower SSI and lower recurrence rates compared with primary suture and PPM, and less seroma compared with PPM^31^. This demonstrates the great potential of biological surgical meshes derived from bovine pericardium, especially those with the addition of antibiotics.

## CONCLUSION

The bovine pericardial mesh impregnated with vancomycin proved to be effective in controlling the infection caused by methicillin-resistant *Staphylococcus aureus in vivo*. The mesh impregnated with silver nanoparticles proved to be inferior and with a high local reaction when used for MRSA infection control. 
